# Addressing the physician burnout epidemic with resilience curricula in medical education: a systematic review

**DOI:** 10.1186/s12909-021-02495-0

**Published:** 2021-02-01

**Authors:** Chanhee Seo, Mario Corrado, Karine Fournier, Tayler Bailey, Kay-Anne Haykal

**Affiliations:** 1 Department of Family Medicine, Faculty of Medicine, University of Ottawa, 451 Ch. Smyth Rd. (2024), Ottawa, ON K1H 8M5 Canada; 2 Health Sciences Library, University of Ottawa, Ottawa, ON K1H 8M5 Canada; 3 Department of Family Medicine, Faculty of Medicine, McMaster University, Hamilton, ON L8P 1H6 Canada

**Keywords:** Systematic review, Resilience, Curriculum, Undergraduate medical education (UGME), Post-graduate medical education (PGME)

## Abstract

**Background:**

A variety of stressors throughout medical education have contributed to a burnout epidemic at both the undergraduate medical education (UGME) and postgraduate medical education (PGME) levels. In response, UGME and PGME programs have recently begun to explore resilience-based interventions. As these interventions are in their infancy, little is known about their efficacy in promoting trainee resilience. This systematic review aims to synthesize the available research evidence on the efficacy of resilience curricula in UGME and PGME.

**Methods:**

We performed a comprehensive search of the literature using MEDLINE, EMBASE, PsycINFO, Educational Resources Information Centre (ERIC), and Education Source from their inception to June 2020. Studies reporting the effect of resilience curricula in UGME and PGME settings were included. A qualitative analysis of the available studies was conducted in accordance with the Preferred Reporting Items for Systematic Reviews and Meta-Analyses (PRISMA) guidelines. Risk of bias was assessed using the ROBINS-I Tool.

**Results:**

Twenty-one studies met the inclusion criteria. Thirteen were single-arm studies, 6 quasi-experiments, and 2 RCTs. Thirty-eight percent (8/21; *n* = 598) were implemented in UGME, while 62 % (13/21, *n* = 778) were in PGME. There was significant heterogeneity in the duration, delivery, and curricular topics and only two studies implemented the same training model. Similarly, there was considerable variation in curricula outcome measures, with the majority reporting modest improvement in resilience, while three studies reported worsening of resilience upon completion of training. Overall assessment of risk of bias was moderate and only few curricula were previously validated by other research groups.

**Conclusions:**

Findings suggest that resilience curricula may be of benefit to medical trainees. Resilience training is an emerging area of medical education that merits further investigation. Additional research is needed to construct optimal methods to foster resilience in medical education.

**Supplementary Information:**

The online version contains supplementary material available at 10.1186/s12909-021-02495-0.

## Background

Medical studies present a unique set of challenges and stressors that reach beyond the academic curriculum. In addition to a substantial workload, medical students and residents are confronted with a wide range of stressors including inflexible work schedules, sleep deprivation, fatigue, time-consuming clerical and administrative responsibilities, insufficient access to allied health personnel and staff (e.g., nurses, social workers), and unwelcoming learning environments [1]. Coupled with societal- and self-expectations [2], medical students and residents are at high risk for compassion fatigue [3–5], low self-esteem [6], decreased mental health [7–13], and overall poor quality of life [14–20]. The implications of elevated psychological distress among medical students and residents are well-documented and include diminished academic accomplishment [21, 22], substance abuse [9, 23–25], decreased empathy [26, 27], increased professional misconduct [28], and suicide [29, 30]. An abundance of systematic reviews have highlighted the significantly negative impact of medical education on student wellness [31–38]. Specifically, a worrying prevalence of burnout has been widely reported among medical students and residents across several specialties and numerous countries including the United States, Qatar, Australia, Canada, New Zealand, France, England, Switzerland, Thailand, India, Germany, Greece, and Trinidad & Tobago [9, 10, 30, 39–47]. Burnout is traditionally defined as a maladaptive response to chronic work stress [48, 49] and characterized by a triad of emotional exhaustion (i.e., loss of enthusiasm), depersonalization (i.e., cynical thought patterns) and a reduced sense of personal accomplishment. Together, they work to negatively impact the well-being of both the medical student and the patient population [50].

The burnout epidemic among medical trainees was only recently acknowledged. In the 1990s, the “Triple Aim” of healthcare was established by healthcare institutions and organizations in the US. This new initiative emphasized patient satisfaction, quality of care and cost reductions [51]. It was not until the 2000s that staff and healthcare provider satisfaction was formally considered in the rebranded “Quadruple Aim” of healthcare [52]. In 2015, physician groups in both the United States and Canada began to adopt formal campaigns recognizing the resident burnout epidemic. In the United States, the Accreditation Council for Graduate Medical Education (ACGME) amended its accreditation requirements to address trainee well-being and resilience more comprehensively across all residency and fellowship programs [53]. In addition to providing self-care resources, the campaign helped residents find meaning in their work, enhanced communication and professional relationships, evaluated and promoted safety in the working and learning environment, and provided education and resources to identify and treat burnout, depression, substance abuse and other challenges [54]. In Canada, the 2015 CanMEDS Physician Competency Framework outlined that, in order to be a professional, one must have a commitment to self [55]. In response, an increasing number of residency programs have restructured their training to provide and foster specific skills and dispositions towards work-life balance and self-care [56, 57].

The ACGME’s formal recognition of the resident burnout epidemic paved the way for new wellness initiatives. Institutions have begun exploring a vast array of health-promotion programs including mindfulness [58–60], yoga [61, 62], self-hypnosis protocols [63], small group debriefing and stress-management programs [64], curricular changes [65], evaluation changes [66, 67], time management programs [68], reflective writing sessions [69, 70], and self-development groups [71]. However, recent literature on therapeutic stress management programs for medical students and residents have revealed mixed and inconsistent results, with overall unclear long-term benefits. In response, medical schools and residency programs have begun exploring alternate wellness interventions, aiming to prevent burnout long-term by fostering trainee resilience [72–74].

Resilience is broadly defined but can be conceptualized as the ability to face adversity forthrightly and intentionally instead of aiming merely to survive through hardships. Resilient individuals re-frame challenges as opportunities for growth and thus willingly engage with the harsh realities of life in a healthy manner that ultimately achieves goals at a minimal physical and psychological cost [75]. A systematic review on resilience identified five themes used to define resilient individuals: rising above adversity, adapting and adjusting, resilience as a dynamic process, “ordinary magic” (i.e., resilience is an inherent trait in all people) and mental illness as a marker of resilience [76]. Resilience was initially regarded as an inherited, static character trait [77, 78], however research has identified it as a dynamic and transient quality [78]. Importantly, resilience education requires a foundation of self-awareness and the ability to self-monitor [75]. This necessitates that the individual willingly accepts their limits and uncertainties, and uses their insight to recognize errors and problem solve [77]. Initial studies have indicated that physicians who exhibit high-resilience personality traits have an objectively-elevated sense of overall well-being, provide better quality patient care and ultimately contribute to an overall decrease in healthcare costs [79].

These initial observations support the implementation of resilience-based interventions in medical training to prepare trainees for the inevitable hardships of clinical practice [75, 80]. Although such interventions have been developed and integrated into medical school and residency programs, little is known about their efficacy in promoting resilience. To address this paucity in the literature, we conducted a systematic review to synthesize the available research evidence on the efficacy of resilience curricula in undergraduate and postgraduate medical education.

## Methods

### Protocol and registration

A systematic review protocol was prospectively developed and registered in the International Prospective Register of Systematic Reviews (PROSPERO, CRD42020191511). This systematic review was performed in concordance with the Cochrane Handbook for systematic reviews of interventions and the Preferred Reporting Items for Systematic Reviews and Meta-Analysis Protocols (PRISMA-P) statement (Additional file 1) [81–83].

### Search method

A systematic search of the literature was performed by an information specialist (K.F.) in MEDLINE(R) ALL (Ovid, 1946 to June 15, 2020), Embase (Ovid, 1947 to 2020 June 15), APA PsycInfo (Ovid, 1806 to June Week 22,020), ERIC (Ovid, 1965 to March 2020), Education Source (EBSCOHost, 1880–2020) from database inception to June 16, 2020 using a combination of subject headings terms and keywords (Additional file 2): “medical education”, “medical student”, “resilience”, “curriculum”, and “training”. In addition, a manual search of the reference lists of the retrieved articles was conducted to capture all relevant studies for potential inclusion.

### Eligibility criteria

We included all peer reviewed primary research articles that implemented resilience curricula in UGME and PGME settings and reported efficacy outcome measures. As per our review protocol published a priori, the inclusion criteria for studies were: (1) both single- and double-arm studies of either qualitative or quantitative nature in which the participants received a clearly defined curriculum which included at least one concept of resilience including, but not limited to, coping skills, self-efficacy, goal-setting, and emotional regulation skills; (2) study participants including undergraduate and postgraduate medical learners; (3) an outcome measure assessing the effect of resilience curricula through quantitative or qualitative research instruments. Both randomized controlled trials and quasi-experimental studies were included. No restrictions were placed on the study subjects’ age, gender, country, level or specialty of training, and length of curricula and follow-up period.

We excluded review articles, editorials, letters, commentaries, theoretical articles (e.g., curricular development), and non-English articles; however, a manual search of the reference lists of review articles was conducted to ensure broad and comprehensive inclusion of the available literature. For the purpose of this review, studies were excluded if their primary focus was therapeutic in nature (e.g., cognitive therapy, counselling, and mind-body skills). Finally, studies that only described participant satisfaction or those that only described the implementation of resilience curricula were also excluded.

### Study selection and data abstraction

After identifying all citations and removing duplicates, a two-step screening process was employed to determine the relevancy of the identified articles. First, each study title and abstract was independently screened for eligibility in accordance with the aforementioned eligibility criteria by two reviewers (C.S. and M.C.). An inter-rater calibration test was performed using 20 randomly identified articles prior to formally commencing the screening process to ensure reliable screening accuracy. Following the title-abstract screening, the same two reviewers independently retrieved the full text of relevant articles and determined their eligibility for inclusion using the preset inclusion criteria. Any discrepancies between the two reviewers were resolved by consensus. When no consensus could be reached, a third reviewer was consulted.

A standardized data collection table was created by the review team prior to formally commencing the extraction process. Extracted variables included: (1) study characteristics (e.g., country of origin, study design, target population, sample size); (2) curriculum description (e.g., name, curricular content, delivery mode, duration and frequency); (3) resilience outcome scales; and (4) secondary curriculum outcome measures. Two reviewers (C.S. and M.C.) independently extracted the data and compared the results for verification. Any disagreements between the two reviewers were resolved by consensus after consulting the entire review team.

### Analysis of studies

A primarily qualitative appraisal of the literature was conducted following the methodological guidelines for qualitative reviews outlined in the Cochrane Handbook [83]. When appropriate, descriptive statistics were utilized to present the overall trend or effect of results. Due to the heterogeneity in both curricula and outcome measures across the included studies, a pooled analysis of the effects of curricula using a meta-analytic methodology was not possible.

### Quality appraisal

A risk of bias assessment of each included study was performed by two independent reviewers (C.S. and M.C.) using the ROBINS-I tool [84, 85]. Risk of bias was assessed across seven domains (confounding, selection, classification, intervention, missing data, measurement, reporting). Results of quality assessments were compared, and when there were any disagreements, the entire review team was consulted which were then resolved by consensus.

## Results

### Study selection

**Fig. 1:**
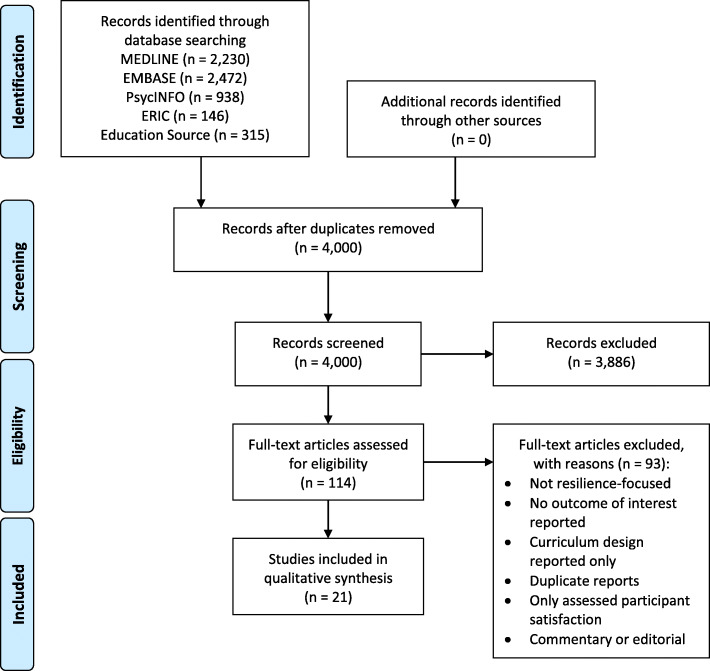
PRISMA flow diagram of the study screening process

Figure 1 depicts the study selection process in a PRISMA flow diagram. The initial electronic search of the databases identified 6101 citations. Two thousand one hundred one duplicate records were removed using Covidence (Veritas Health Information, Melbourne, Australia), which left 4000 references for the screening phase. Following the title-abstract screening, 3886 studies were deemed to be irrelevant for the present review based on the exclusion criteria. Inter-rater reliability for the title-abstract screening was excellent (k > 0.90). Of the 114 studies assessed for eligibility in the full-text screening, 93 studies were further excluded as they did not meet the inclusion criteria. No additional studies were identified after a manual screening of reference lists of the included studies. In total, 21 studies that fully satisfied the previously stipulated eligibility criteria were included in this review [57, 86–105].

### Study characteristics

**Fig. 2:**
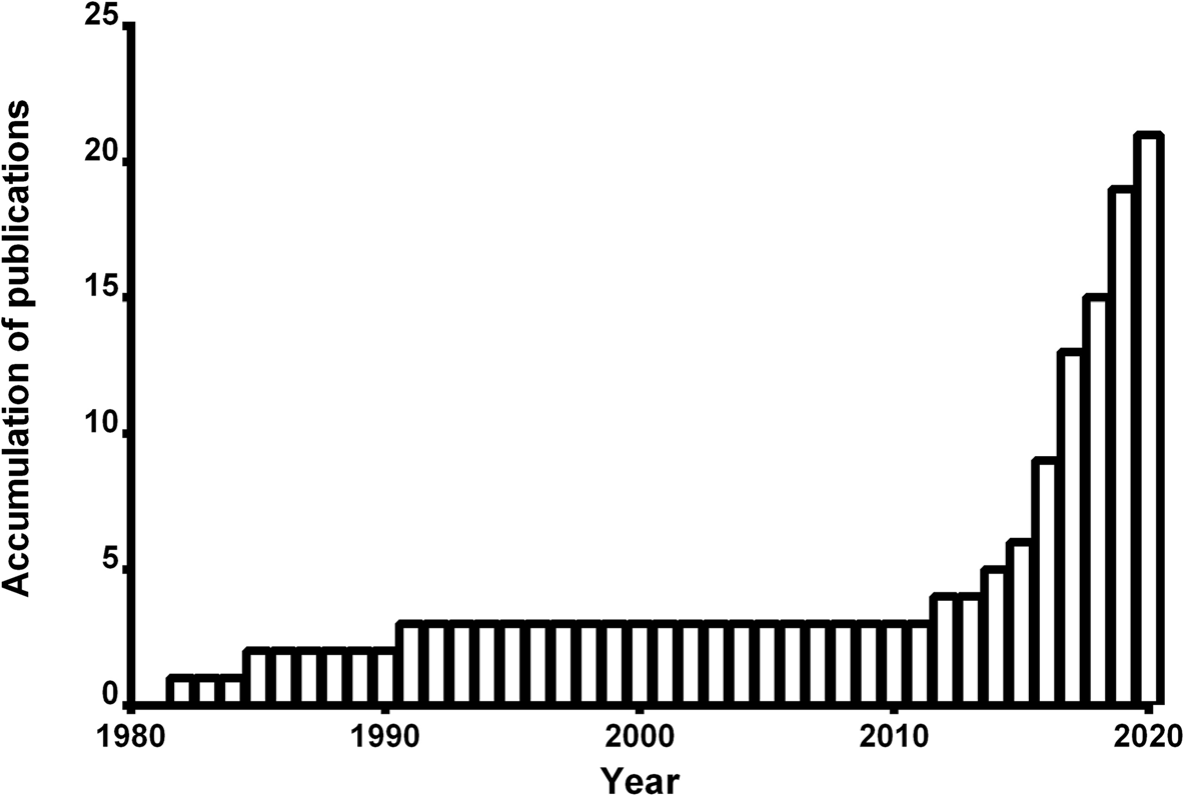
Number of accumulated publications over time

The characteristics of the included studies are collated and summarized in Table 1. Of the 21 studies included in this review, 13 studies (62%, *n* = 984 total participants) used a single-arm design that evaluated the effect of resilience curricula pre- and post-intervention, 2 studies (9.5%, *n* = 100) used a randomized controlled design, and 6 (28.5%, *n* = 292) used a quasi-experimental design. Sample sizes of studies varied between 12 and 258, but none of the studies performed an a priori sample size determination. As shown in Fig. 2, the earliest study on resilience curricula in medical education was published in 1982, with the majority of studies (90.5%, 19/21) published in the last decade.

**Table 1:** Study characteristics and outcomes reported

Reference; Country	Study design; Population; n (intervention): n (control)	Name of curriculum	Curriculum objectives	Facilitator	Training methods	Frequency; Duration	Follow-up	Attrition Rate	Effect of curriculum on outcome measures
Bird et al., 2017 [94]; USA	Prospective cohort, single-arm; 1st-year IM residents; 81 participants	Not specified	Goal setting, managing expectations, processing stressful clinical events, identifying sources of gratitude	Chief resident	60- to 90-min sessions consisting of a didactic introduction followed by small group discussion, reflection, and skill-building exercises (e.g., reflective writing)	4 sessions over one year	Pre- and post-intervention	25% (20 of 81) pre-intervention, 21% (17 of 81) post-intervention.	Connor-Davidson Resilience Scale: 72.5 + 10.2 pre-intervention vs. 68.6 + 10.1 post-intervention (*p* = 0.034). Non-proprietary single item burnout measure: 1 out of 28 participants with symptoms of burnout pre-intervention vs. 8 of 25 post-intervention (*p* = 0.009). 84% of participants (54 of 64) stated they feel more callous toward people post-intervention vs. 59% (36 of 61) in pre-intervention (*p =* 0.002). Curriculum feedback: 75% of participants (48 of 64) supported continuation of the program, 70% stated increased comfort for discussing stress and burnout, 64% reported having the necessary stress and burnout management skills.
Brennan et al., 2016 [92]; USA	Prospective cohort, single-arm; 1st-year medical students; 42 participants	Not specified	Building relaxation skills, mindfulness meditation, adaptive coping skills, balancing life and school, positive psychology, basic nutrition	Physician, psychologist, or counsellor	Each session included educational and skill building exercises. Participants were given written worksheets and volunteered personal examples for discussion. Sessions concluded with a 10 min relaxation exercise.	8 sessions over one academic year (i.e., Sept-May; 4 per semester)	Pre- and post-intervention	26% (11 of 42)	Beck Depression Inventory-II: 7.7 + 4.8 pre-intervention vs. 7.5 + 6.3 post-intervention (*p* > 0.05). Beck Anxiety Inventory: 9.9 + 6.3 pre-intervention vs. 6.7 + 5.3 post-intervention (*p* = 0.016). Coping Self Efficacy Scale: 164.3 + 25.2 pre-intervention vs. 182.9 + 35.8 post-intervention (*p* = 0.001). Self-reported perceived relaxation: 1.6 + 0.5 pre-intervention vs. 2.5 + 0.7 post-intervention (*p* < 0.001).
Brennan et al., 2019 [100]; USA	Prospective cohort, double-arm (quasi experiment); FM residents; 19:13	Not specified	Identifying personal and professional values, improving self-awareness, balancing and prioritizing professional and personal time, using values to improve time management, maintaining supportive professional and personal relationships, and learning effective relaxation strategies	Healthcare professionals	Interactive, skill-based sessions containing handouts and short didactic presentations. Sessions concluded with a skill-based exercise such as breathing awareness, mindfulness meditation, basic relaxation, imagery, or progressive relaxation.	8 h of programming over one year	Pre- and post-intervention and after 1, 2, and 3 years	1 of 19 in intervention group, 3 of 13 in control group after 1st year; 3 of 17 in intervention group after 2nd year; 0 of 8 in intervention after 3rd year.	*Immediate post-intervention* Maslach Burnout Scale: EE: 13.5 + 9.6 intervention vs. 21.4 + 12.6 control (*p* < 0.05); DP: 3.4 + 3.1 intervention vs. 7.0 + 4.1 control (*p* < 0.05); PA: 35.67 + 7.7 intervention vs. 33.20 + 9.0 control (*p* > 0.05); Resilience: 79.06 + 10.72 intervention vs. 72.75 + 22.3 control (*p* > 0.05). Professional Quality of Life Scale: BO: 19.44 + 5.28 intervention vs. 21.8 + 7.44 control (*p* > 0.05); STS: 21.61 + 5.18 intervention vs. 24.60 + 4.97 control (*p* > 0.05); CS: 41.06 + 5.80 intervention vs. 39.20 + 8.56 control (*p* > 0.05). *1-Year follow-up (2nd year post-intervention; intervention group only)* Maslach Burnout Scale: EE: 15.4 + 9.9 vs. 13.9 + 9.1 baseline (*p* > 0.05); DP: 4.5 + 4.2 vs. 5.2 + 4.1 baseline (*p* > 0.05); PA: 39.6 + 5.9 vs. 36.1 + 7.7 baseline (*p* > 0.05); Resilience: 80.1 + 10.7 vs. 77.3 + 12.2 baseline (*p* > 0.05). Professional Quality of Life Scale: BO: 19.3 + 5.2 vs. 20.7 + 5.9 baseline (*p* > 0.05); STS: 21.6 + 4.8 vs. 23.3 + 6.6 baseline (*p* > 0.05); CS: 41.5 + 4.8 vs. 40.8 + 6.4 baseline (*p* > 0.05). *2-Year follow-up (3rd year post-intervention; intervention group only)* Maslach Burnout Scale: EE: 17.5 + 11.0 vs. 13.9 + 9.1 baseline (*p* > 0.05); DP: 3.5 + 3.7 vs. 5.2 + 4.1 baseline (*p* > 0.05); PA: 36.8 + 8.2 vs. 36.1 + 7.7 baseline (*p* > 0.05); Resilience: 82.1 + 14.9 vs. 77.3 + 12.2 baseline (*p* > 0.05). Professional Quality of Life Scale: BO: 18.0 + 6.7 vs. 20.7 + 5.9 baseline (*p* > 0.05); STS: 19.5 + 5.8 vs. 23.3 + 6.6 baseline (*p* > 0.05); CS: 41.9 + 5.8 vs. 40.8 + 6.4 baseline (*p* > 0.05).
Chaukos et al., 2018 [98]; USA	Prospective cohort, single-arm; IM and PSYC interns; 75 participants	Stress Management and Resiliency Training Program for Residents (SMART-R)	Developing mind-body techniques, stress awareness skills, meaningful goal-setting techniques, positive perspective-taking approaches	Residents	Group sessions utilizing a resident curriculum workbook and corresponding instructor manual.	Three group-based sessions totalling 6 h over 6 months	Pre- and post-intervention	59% (44 of 75)	Maslach Burnout Inventory: EE: 19.0 + 11.1 pre-intervention vs. 29.0 + 12.2 post-intervention (*p* = 0.000017); DP: 8.9 + 6.6 pre-intervention vs. 13.5 + 7.9 post-intervention (*p* = 0.0000058); PA: 37.3 + 6.3 pre-intervention vs. 36.2 + 7.0 post-intervention (*p* = 0.3). Perceived Stress Scale 10-item: 15.7 + 7.0 pre-intervention vs. 17.8 + 6.3 post-intervention (*p* = 0.07). PHQ-9: 3.7 + 4.9 pre-intervention vs. 7.4 + 6.0 post-intervention (*p* = 0.001). FACIT: 12.0 + 9.5 pre-intervention vs. 21.9 + 12.5 post-intervention (*p* = 8.4 × 10^-6^). PSWQ: 48.2 + 11.7 pre-intervention vs. 45.5 + 13.5 (*p* = 0.25). MOCS-A: 24.9 + 8.2 pre-intervention vs. 26.4 + 7.8 post-intervention (*p* = 0.184). SEQS: 31.3 + 3.4 pre-intervention vs. 31.0 + 4.3 post-intervention (*p* = 0.57). LOT-R: 16.0 + 4.24 pre-intervention vs. 15.1 + 5.3 post-intervention (*p* = 0.047). CAMS-R: 31.2 + 7.0 pre-intervention vs. 30.7 + 5.8 post-intervention (*p* = 0.56). IRI-PT: 17.3 + 4.3 pre-intervention vs. 18.4 + 4.2 post-intervention (*p* = 0.09).
Dyrbye et al., 2017 [95]; USA	Prospective cohort, single-arm; 1st-year medical students; 54 (cohort 1^a^), 51 (cohort 2^a^)	SMART	Developing intentional attention and awareness, positive perspective-taking approaches, compassion, personal and professional goal-setting, forgiveness skills, creating and nurturing meaningful relationships	Social worker, psychologist, physicians	Didactic teaching and facilitated small group discussion with skill-based learning (e.g., journaling, reflective exercises).	Eight group-based sessions totalling 12 h (cohort 1) or 10 h (cohort 2) over one year	Pre- and post-intervention	18.5% (10 of 54; cohort 1), 57% (29 of 51; cohort 2)	*Cohort 1* Maslach Burnout Inventory: 14.6% of participants experienced burnout pre-intervention vs. 28.2% post-intervention (*p* = 0.11). SF-8: Mental QOL: 51.2 + 7.3 pre-intervention vs. 45.57 + 10.61 post-intervention (*p* < 0.001); Physical QOL: 53.0 + 5.9 pre-intervention vs. 52.81 + 6.78 post-intervention (*p* = 0.85). Perceived Stress Scale: 11.1 + 5.8 pre-intervention vs. 15.32 + 5.56 post-intervention (*p* < 0.0001). Connor Davidson Resilience Scale: 6.7 + 1.2 pre-intervention vs. 6.2 + 1.58 post-intervention (*p* = 0.05). Happiness and Gratitude Scale: 5.3 + 0.9 pre-intervention vs. 4.99 + 0.79 post-intervention (*p* = 0.02). IRI: Cognitive: 22.2 + 3.4 pre-intervention vs. 20.56 + 3.87 post-intervention (*p* < 0.01); Emotive: 20.1 + 4.0 pre-intervention vs. 18.03 + 4.46 post-intervention (*p* < 0.01). *Cohort 2* Maslach Burnout Inventory: 17.8% of participants experienced burnout pre-intervention vs. 41.6% post-intervention (*p* = 0.13). SF-8: Mental QOL: 48.7 + 8.8 pre-intervention vs. 43.55 + 8.1 post-intervention (*p* = 0.015); Physical QOL: 54.5 + 5.0 pre-intervention vs. 52.69 + 3.9 post-intervention (*p* = 0.14). Perceived Stress Scale: 11.2 + 6.0 pre-intervention vs. 14.82 + 7.1 post-intervention (*p* < 0.003). Connor Davidson Resilience Scale: 6.6 + 1.3 pre-intervention vs. 6.5 + 0.9 post-intervention (*p* = 0.79). Happiness and Gratitude Scale: 5.3 + 1.2 pre-intervention vs. 4.9 + 0.7 post-intervention (*p* = 0.01). IRI: Cognitive: 21.9 + 3.0 pre-intervention vs. 21.0 + 3.0 post-intervention (*p* = 0.18); Emotive: 21.0 + 3.6 pre-intervention vs. 20.14 + 3.4 post-intervention (0.26).
Forbes et al., 2020 [104]; Australia	Prospective cohort, double-arm (quasi experiment); 1st-year interns; 24:29	Resilience on the Run (RoR)	Understanding mindfulness and self-awareness, practical mindfulness exercises, effective and healthier interpersonal communication strategies, role-play scenarios for managing difficult work-related situations, understanding burnout and compassion fatigue and their risk factors, information to enhance peer support	Psychiatrist	90-min group sessions focusing on interactive real life scenarios with integrated mindfulness exercises.	4 sessions during second half of intern year	Pre- (T0) and post-intervention (T1) and after 3 months (T2)	4 of 24 in intervention group, 5 of 29 in control group immediately post-intervention; 4 of 24 in intervention group, 8 of 29 in control group after 3 months.	ProQOL^b^: # of participants with compassion fatigue (score < 44): 5 intervention vs. 9 control at T0; 4 vs. 7 at T1; 5 vs. 3 at T2; # of participants with burnout (score > 56): 7 intervention vs. 5 control at T0; 5 vs. 6 at T1; 3 vs. 5 at T2; # of participants with secondary traumatic stress (score > 56): 4 intervention vs. 7 control at T0; 4 vs. 7 at T1; 2 vs. 8 at T2. K10^b^: T0: 19.13 intervention vs. 16.97 control; T1: 19.2 intervention vs. 17.83 control; T2: 19.35 intervention vs. 17.05 control.
Holtzworth-Munroe et al., 1985 [87]; USA	Randomized controlled trial; 1st and 2nd-year medical students; 20:20	Not specified	Recognizing and changing maladaptive cognitions that precipitate stressful feelings, developing meditation and progressive muscle relaxation techniques as coping mechanism.	Doctoral student in clinical psychology	1-h group sessions focusing on developing and practicing coping skills involving cognitive restructuring, meditation, and progressive muscle relaxation.	6 weekly meetings	Pre- and post-intervention and after 10 weeks	2 of 20 in intervention group, 4 of 20 in control group immediately post-intervention; 5 of 20 in intervention group, 11 in control group after 10 weeks.	Spielberger Trait Anxiety Inventory: Post-intervention: No significant difference between intervention vs. control groups in anxiety before test, anxiety during test, general worry, nervousness, social anxiety, weekly tension, weekly depression, trait anxiety, and self-esteem; 10-week follow-up: Significant improvement for intervention group seen in anxiety before test measure (*p* < 0.005). No significant difference in other domains. Non-proprietary questionnaires: Compared to control, the intervention group reported greater awareness of their tension (*p* < 0.001 at post-intervention and 10-wk follow-up) and better management of school stress (*p* < 0.04 at post-intervention and 10-wk follow-up).
Kelly et al., 1982 [86]; USA	Prospective cohort, double-arm (quasi experiment); 1st, 2nd, 4th-Year medical students (80%), residents and nurses (20%); 34:14	Not specified	Understanding causes and consequences of stress, relaxation training (i.e., deep-muscle relaxation, relaxing imagery), priority-setting, schedule-planning, time management techniques, cognitive modification skills to decrease stress.	Clinical psychologist, psychology residents, and psychology graduate student	60- to 90-min seminar sessions with didactic presentations, scenario-based group discussions, and stress reduction exercises.	6 sessions over 3 weeks	Pre- and post-intervention	None	Stress Knowledge Inventory: 21.7 + 2.2 intervention vs. 16.6 + 2.7 control (*p* < 0.0001). Jenkins Activity Schedule (for stress level): Jenkins Type A Scale: 227.2 + 63.8 intervention vs. 252.0 + 66.6 control (NS); Jenkins Speed/Impatience Scale: 149.6 + 50.7 intervention vs. 154.5 + 72.1 control (NS); Jenkins Hard Driving Scale: 97.8 + 27.1 intervention vs. 116.4 + 38.3 control (*p* < 0.1). Spielberger State-Trait Anxiety Inventory: 38.6 + 9.3 intervention vs. 42.8 + 8.9 control (NS). Stressful Situations Rating: 53.3 + 18.2 intervention vs. 71.2 + 11.4 control (*p* < 0.005).
McCue et al., 1991 [88]; USA	Prospective cohort, double-arm (quasi experiment); IM, IM/PEDS & PEDS residents; 43:21	Not specified	Developing interpersonal and relationship skills, task prioritization, techniques to enhance stamina and attend to self-care needs, understanding and preventing maladaptive coping responses, positive perspective taking.	Not specified	4-h group workshop with didactic lectures, group discussions, self-reflection, videotaped vignettes to stimulate discussion on coping under stressful situations, role-playing, experiential exercises.	One time	Pre- and post-intervention after 6 weeks	None	ESSI Stress Systems Instrument: Composite mean score increased from 16.67 at baseline to 17.94 at 6 wks after workshop for intervention group vs. 16.72 to 16.07 for control group (*p* < 0.001). Significant improvement seen in self-care (*p* < 0.05), support seeking (*p* < 0.01), behavioural stress symptoms (*p* < 0.01), and emotional stress symptoms (*p* < 0.05). Maslach Burnout Scale (reported in net change): EE: 1.23 intervention vs. -3.38 control; DP: -0.26 intervention vs. -1.09 control; PA: -0.58 intervention vs. -0.57 control.
Orr et al., 2019 [101]; USA	Prospective cohort, single-arm; IM residents; 17 participants	Fostering Resilience through Art in Medical Education (FRAME)	Increasing awareness of the importance of thoughtful and flexible thinking, practicing relational communication skills, identifying difficult or stressful aspects of work, reflecting on important, meaningful, or satisfying aspects of work.	Art museum instructors, IM faculty facilitators	4-h workshop with interactive activities involving artwork at the Philadelphia Museum of Art. After each activity, participants reflect on how the experience can be applied to medical or clinical environment.	One time	Pre- and post-intervention and after 3 months	0 of 17 in intervention group post-intervention; 9 of 17 in intervention group after 3 months.	Maslach Burnout Scale: EE: 3.75 + 1.39 pre-intervention vs. 3.00 + 1.72 post-intervention (at 3 mo); DP: 3.49 + 1.80 pre-intervention vs. 2.70 + 1.94 post-intervention (at 3 mo); PA: 4.77 + 1.00 pre-intervention vs. 5.15 + 0.92 post-intervention (at 3 mo).
Peng et al., 2014 [90]; China	Prospective cohort, double-arm (quasi experiment); Medical students (year not specified); 30:30	Penn Resilience Program (PRP)	Connecting thoughts and emotions, challenging irrational thoughts and beliefs, cognitive training, self-confidence and interpersonal communication, coping strategies, behavior modification exercises and problem-solving exercises	Not specified	90 to 120-min group sessions involving discussion and experience sharing among participants. Minimal didactic portion.	10 weekly sessions	Pre- and post-intervention	None	High-Resilience group: The positive emotion scores significantly increased, total negative emotion and expression suppression scores decreased significantly after training. Low-Resilience group: Resilience, positive emotion and cognitive appraisal scores increased significantly, their negative emotion and expression suppression decreased significantly after training. Follow-up interviews: 90% reported reduced negative emotions and increased positive emotions. 87% showed that they had learned different coping strategies for various complicated situations.
Pereira et al., 2015 [91]; Brazil	Prospective cohort, single-arm; 2nd, 3rd, 4th year medical students; 76 participants	Strategies of Coping with Professional Stress	Understanding stress and coping strategies, course-related stressors, psychological distress, concept of quality of life, stress coping strategies, medical career, personality and resilience, ego defence mechanisms, work psychodynamics and cognitive restructuring.	Professors/Researchers	Mix of didactic presentations and activity-based sessions. Sessions incorporated Jacobson’s progressive relaxation practice.	Fortnightly for 4 months	Pre- and post-intervention	None	45% were able to better manage their time, 57% better able to communicate and express themselves and 76% pay more attention to their feelings after taking course. 68% were more assertive in their friend and family relationships and 75% respected people and the differences between people more than before the elective. 66% had better nutrition, 83% increased self-reflection and reflection of their desires, 50-60% were more tolerant of frustration, limitations and of others. 67% reported less stress symptoms, 76% incorporated new coping strategies and 26% saw challenges in a more positive light, leading to less stress.
Riall et al., 2018 [99]; USA	Prospective cohort, single-arm; 1st-5th year surgery residents; 49 participants	Energy Leadership Well-Being and Resiliency Program	Energy Leadership, team building, communication, work-life integration, goal setting, empathy, strategic diet and exercise, posture for the surgeon/ergonomics, stress-reduction techniques and mindfulness/meditation.	Not Specified	Activity-based group experiential workshops	Monthly over the course of one year	Pre- and post-intervention	1/49 (ELI), 9/49 (Beck Depression Inventory and Perceived Stress Scale), 10/49 (Maslach Burnout Inventory), 8/49 (PWBI).	ELI: 3.16 + 0.24 pre-intervention vs. 3.24 + 0.32 post-intervention (*p* = 0.03). Perceived Stress Scale: 17.0 + 7.2 pre-intervention vs. 15.7 + 6.2 post-intervention (*p* = 0.05). Beck Depression Inventory: 7.43 + 7.9 pre-intervention vs. 3.24 + 0.32 post-intervention (*p* = 0.03). Maslach Burnout Inventory: EE: 16.8 + 8.4 pre-intervention vs. 14.4 + 8.5 post-intervention (*p* = 0.04); Professional efficacy: 27.8 + 6.9 pre-intervention vs. 29.8 + 5.9 post-intervention (*p* = 0.09); Cynicism: 10.31 + 7.9 pre-intervention vs. 12.0 + 8.6 post-intervention (*p* = 0.21). PWBI: 3.2 + 0.24 pre-intervention vs. 3.2 + 0.32 post-intervention (*p* = 0.77).
Rogers et al., 2016 [93]; South Africa	Prospective cohort, single-arm;Final-year BCMP students; 62 participants	Educational Interventions to Enhance Personal Resilience	Understanding the importance of resilience for healthcare professionals.	Not specified	90-min workshop with a mix of didactic teaching, multimedia presentation, small group discussion and problem-solving, and reflection.	One time	Pre- and post-intervention after 8 weeks	31% (19 of 62)	Connor-Davidson Resilience Scale: 77.37 (95% CI 75.53-79.81) pre-intervention vs. 74.12 (95% CI = 70.79-77.45) post-intervention (*p* = 0.38).
Runyan et al., 2016 [57]; USA	Prospective cohort, single-arm; 2nd-year FM residents; 12 participants	Physician as Leader	Understanding mindfulness and its relevance in physician wellness and patient outcomes, mental focusing strategies, increasing self-awareness, identifying personal values, developing narrative/appreciative thinking and personal tools for maintaining resiliency, preventing burnout and enhancing empathic capacity.	Behavioural science faculty member	2 h session with a mix of didactic lectures, multimedia presentations, reflective-writing exercising/journaling, worksheets activities, meditation exercises, and group discussion	4 weekly sessions	Pre- and post-intervention after 3 months	3 of 12 in pre-intervention, 0 of 12 in post-intervention	Self-Compassion Scale: Self-kindness: 5.67 + 1.66 pre-intervention vs. 6.56 + 0.73 post-intervention; Self-judgment: 6.22 + 2.17 pre-intervention vs. 5.33 + 0.87 post-intervention; Common humanities: 5.89 + 1.36 pre-intervention vs. 6.56 + 1.88 post-intervention; Isolation: 6.11 + 1.62 pre-intervention vs. 6.67 + 1.73 post-intervention; Mindfulness: 6.67 + 1.73 pre-intervention vs. 8.11 + 1.69 post-intervention; Over-identified: 4.78 + 2.17 pre-intervention vs. 5.44 + 1.51 post-intervention; Overall self-compassion: 35.33 + 6.23 pre-intervention vs. 38.67 + 4.82 post-intervention. Maslach Burnout Inventory: EE: 20.44 + 9.36 pre-intervention vs. 18.00 + 9.88 post-intervention; Professional efficacy: 24.78 + 7.68 pre-intervention vs. 26.89 + 4.29 post-intervention; Cynicism: 15.67 + 8.94 pre-intervention vs. 15.33 + 8.07 post-intervention. Perceived Stress Scale: 18.11 + 6.70 pre-intervention vs. 14.78 + 7.05 post-intervention. Jefferson Empathy Scale: 110.56 + 18.30 pre-intervention vs. 122.11 + 5.49 post-intervention.
Saadat et al., 2012 [89]; USA	Randomized control trial; 1st-3rd year Anesthesiology residents; 20:20:20, each in Wellness Intervention Group (WIG), No-treatment control group with release time (NTC-RT), and No-treatment control group with routine duties (NTC-RD).	Coping with Work and Family Stress	Identifying stressful situations, practicing effective problem-solving and communication skills, modifying cognitive and appraisal processes, stress management, preventing avoidance coping mechanisms.	Not Specified	1.5-h sessions incorporating didactic learning materials, group discussions, self-reflection and stress management exercises.	16 weekly sessions	Pre- and post-intervention	1 of 20 in WIG; 1 of 20 in NTC-RD group.	WIG vs. NTC-RD: Significant decrease in perceived stress and anxiety, significant increase in problem solving and social support from work. Near-significant decrease in perceived stress as a spouse/partner, avoidance, somatic symptoms and depression for the WIG group compared to the NTC-RD group. NTC-RT vs WIG: Significant increase in social support from work. Near-significant decrease in total alcohol consumption and avoidance for the WIG group compared to the NTC-RD group. NTC-RD vs NTC-RT: Significant increase in problem solving. Near-significant decrease in somatic symptoms and perceived stress as a parent for the NTC-RT group compared to the NTC-RD group.
Saint Martin et al., 2019 [102]; USA	Prospective cohort, single-arm; 1st-4th year Pathology residents; 17 participants	Not specified	To gain knowledge on stress management and burnout in health care, and improve self-care, group care, and patient care.	Faculty wellness advisor	60-min wellness talk sessions involving self-reflection, didactic presentations, team building activities, discussing stressful situations and encounters with patients or faculty members, and mindfulness exercises.	12 monthly sessions	Pre- and post-intervention	24% (4 of 17)	54% reported increased knowledge about causes of burnout, and improvement by 114, 144, and 166% was seen in knowledge of personal, group, and institutional resources, respectively. Significant (*p* = 0.00001) improvement in personal wellness knowledge.
Shapiro et al., 2019 [103]; USA	Prospective cohort, single-arm; IM Residents; 258 participants	Call to Wellness spiritual care curriculum	To build resilience and promote well-being among physician trainees, understand depression and burnout, and provide resources and support.	Representatives from Hospital Spiritual Care (HSC), including a chaplain and spiritual care staff.	60-min individual or group session with HSC representatives where participants openly discussed religious, cultural, and/or spiritual beliefs. Sessions also focused on resident stressors, resources for coping, and building resilience	One time	Pre- and post-intervention	None	Pre-intervention: 24% rated their overall well-being as negative, 51% as neutral, 25% as slightly positive, positive or very positive. Post-intervention: 24% rated their overall well-being as negative, 25% as neutral, 51% as slightly positive, positive or very positive (*p* < 0.001).
Slavin et al., 2017 [96]; USA	Prospective cohort, double-arm (quasi-experiment); 1st-year Pediatric residents; 17:18	Not specified	Managing stress and finding meaning, learning resilience topics and tools, developing effective communication skills, learning how to better cope with stresses inherent in residency, and enhancing interactions with residency program leadership.	Medical faculty members	2-h group workshop involving didactic teaching followed by individual session with a faculty member on the topic of communication with colleagues and staff. A third faculty member met with the chief residents and residency directors regularly (approx 1/month with follow up by email) to try to help them reduce unnecessary stressors and enhance communication with residents.	One-time workshop and 1 h session every month or every other month	Pre- and post-intervention	None	Maslach Burnout Inventory: EE: 21.8 + 8.1 intervention vs. 29.6 + 9.3 control (*p* < 0.05); DP: 6.4 + 3.9 intervention vs. 10.2 + 4.2 control (*p* < 0.01). State-Trait Anxiety Inventory: 42.8 + 6.7 intervention vs. 50.8 + 8.3 control (*p* < 0.01). Center for Epidemiologic Studies Depression Scale: 13.9 + 9.7 intervention vs. 21.2 + 12.8 control (*p* = 0.07)
Song et al., 2020 [105]; USA	Prospective cohort, single-arm; Surgery residents; 25 participants	Not Specified	To help participants improve resilience, well-being, and engagement at work, while reducing burnout.	Professional resilience coach	2 h group workshop followed by regular 1 h individual coaching sessions catered to each intern’s needs.	One group workshop and 8 individual coaching sessions across academic year.	Pre- and post-intervention	All participants completed the quantitative assessments. 9/25 did not participate in semi-structured interviews.	Brief Resilience Scale: 3.8 + 0.8 pre-intervention vs. 4.2 + 0.7 post-intervention (*p* = 0.002). Abbreviated Maslach Burnout Inventory: No significant changes in the AMI scores or the proportion of interns at risk of burnout before (60%) and after (52%) coaching (*p* = 078). Scale of Positive and Negative Experience: 6.7 + 8.2 pre-intervention vs. 8.4 + 8.3 post-intervention (*p* = 0.14).
Tucker et al., 2017 [97]; Canada	Prospective cohort, single-arm; 3rd-year medical students; 165 participants	Compassion Fatigue Program	To provide students an opportunity to self-reflect and understand ways of recognizing and reducing compassion fatigue and burnout.	Trained compassion fatigue educators	Group workshop incorporating didactic learning material, self-reflection and assessment exercises, and burnout and compassion fatigue reduction exercises.	One workshop	Pre-intervention (August); Time 2 (January); Time 3 (April)	106/165 pre-intervention, 147/165 at Time 2; 133/165 at Time 3	ProQOL: Compassion satisfaction: 39.96 (95% CI 38.63-41.29) baseline vs. 39.39 (37.11-41.67) Time 2 vs. 34.84 (33.13-36.55) Time 3 (*p* < 0.05); Burnout: 22.07 (20.81-23.34) baseline vs. 23.67 (21.47-25.85) Time 2 vs. 26.31 (24.67-27.95) Time 3 (*p* < 0.05); Secondary Traumatic Stress: 20.37 (18.85-21.88) baseline vs. 21.06 (18.47-23.64) Time 2 vs. 20.75 (18.81-22.69) Time 3.

^a^ Curriculum was administered to both 2014 matriculates (cohort 1) and 2015 matriculates (cohort 2). Only data from individual cohorts were reported

^b^ No statistical test was performed due to small sample size at each time point

Abbreviations: *BCMP* Bachelor of Clinical Medical Practice; *BO* Burnout; *CAMS-R* Cognitive and Affective Mindfulness Scale; *CF* Compassion fatigue; *CS* Compassion satisfaction; *DP* Depersonalization; *EE* Emotional exhaustion; *ELI* Energy Leadership Index; *FACIT* Functional Assessment of Chronic Illness Therapy-Fatigue Scale; *IM* Internal Medicine; *IRI-PT* Interpersonal Reactivity Index Perspective-Taking subscale; *K10* Kessler Psychological Distress Scale; *LOT-R* Revised Life Orientation Test; *MOCS-A* Measure of Current Status-Part A; *NS* Not significant; *PA* Personal accomplishment; *PHQ-9* Patient Health Questionnaire 9 item (for clinical depression); *ProQOL* Professional Quality of Life Scale (Version 5); *PSWQ* Penn State Worry Questionnaire; *PWBI* Physician Well-Being Index; *SEQS* Self-Efficacy Questionnaire Scale; *SF* Medical Outcomes Study Short Form (for quality of life); *STS* Secondary traumatic stress

### Study populations

Thirty-eight percent (8/21, *n* = 598 total participants) of the included studies implemented their resilience curricula in a UGME setting: 3 studies were implemented in pre-clerkship, 2 in clerkship, 2 in both pre-clerkship and clerkship, and 1 study did not specify the level of UGME participants. Sixty-two percent (13/21, *n* = 778) implemented the curricula in a PGME setting. Of the studies that tested their curricula among residents, 5 were in internal medicine, 2 in surgery, 2 in family medicine, 2 in pediatrics, 1 in psychiatry, 1 in anesthesiology, 1 in pathology, and 1 in internship. Geographic distribution of the included studies was heavily concentrated in the United States (*n* = 16), with only 1 study in Canada, China, Brazil, South Africa and Australia, respectively.

### Training methods

Considerable heterogeneity existed in the duration and frequency of resilience curricula and ranged from a 1-h single session to 1.5-h weekly sessions for 16 consecutive weeks. 76% (*n* = 16) of the studies implemented in-person training over at least 4 sessions. The majority of studies incorporated more than one component to facilitate resilience, including didactic teaching sessions (*n* = 16) and skill-building exercises around the themes of mindfulness, relaxation, and stress reduction (*n* = 16). Of the 5 resilience curricula that were based on previously developed training models, 2 used the Stress Management and Resilience Training Program [95, 98], and 3 used the Penn Resilience Program [90], Coping with Work and Family Stress Intervention [89], and Energy Leadership Well-Being and Resiliency Program [99], respectively. Two studies implemented non-conventional, outside-of-classroom curricular design. In a study by Shapiro et al. (2019), the authors sought to promote emotional well-being and resiliency in graduate medical trainees through individualized or group spiritual care sessions wherein residents were given an opportunity to openly discuss their religious, cultural, and spiritual beliefs with Hospital Spiritual Care representatives. In another study by Orr et al. (2019), senior internal medicine residents participated in a single, 4-h workshop at the Philadelphia Museum of Art that focused on the medical humanities and artful thinking exercises to enhance resilience and ameliorate burnout. There was a considerable variety in the background of instructors which included residents, psychologists, social workers, chaplain, and staff physicians. Only two studies were facilitated by trained resilience instructors [97, 105].

### Outcome measures

All studies relied on self-report questionnaires to assess curricular outcomes. The majority of studies evaluated the effects of the resilience curriculum using previously validated outcome instruments (90.5%, 19/21), while 2 studies used a new or non-proprietary measurement tool [91, 102]. Most commonly used outcome measure was Maslach Burnout Scale (9/21), followed by Connor-Davidson Resilience Scale (4/21), Perceived Stress Scale (4/21), Professional Quality of Life Scale (3/21), and Spielberger Trait Anxiety Inventory (3/21). Eleven studies assessed the effects of the curriculum immediately pre- and post-intervention with no follow-up. Only 4 studies reported long-term effects of their curriculum (i.e., after at least 12 months of delivering the curriculum).

As shown in Table 1, there was considerable heterogeneity in the efficacy of resilience curricula. While most studies suggested a modest improvement in their efficacy measures upon the completion of resilience curricula, a few studies reported no significant impact, and in some, even worsening of resilience and associated measures. Interestingly, despite positive feedback from students who also supported continuation of the resilience program, Bird et al. (2017) reported that both resilience and burnout measures significantly worsened post-intervention. Similarly, Chaukos et al. (2018) reported significant worsening of the EE and DP scores in the Maslach Burnout Inventory after administration of resilience curriculum and Dyrbye et al. (2017) reported an increasing trend of burnout on the Maslach Burnout Inventory in study participants. Due to the mixed and inconsistent outcome measures reported in the included studies, the overall benefit (or harm) of resilience curricula cannot be determined.

### Feasibility and acceptability

Participant satisfaction and acceptability of resilience curricula were reported in 12 of the 21 studies [87, 88, 91, 94, 95, 97–99, 101, 103–105]. Participants reported generally positive experience and acceptability of resilience curricula, however, the methodology of program assessment was largely heterogeneous and unsystematic.

In Pereira et al. (2015), 90% of the participants reported that the course was helpful, and 76.3% indicated that they had incorporated new coping mechanisms into daily life as a result of the training. Similarly, in Chaukos et al. (2018), 87% considered the resilience skills taught during training to be helpful. While some participants suggested that resilience training should be part of mandatory curricula [91], others found it counterproductive and unnecessary [95].

In terms of curriculum duration, some participants indicated that resilience curricula should be implemented across all years of residency, as resilience skills and techniques may be more instrumental in later years with added stressors and clinical responsibilities [105]. Similarly, 75% of participants stated that the resilience curricula should continue in the following year [94]. 19% reported that training sessions were too short in duration and infrequent [98]. Need for greater availability of resilience training and resources was noted by Tucker et al. (2017) as the authors quote responses from two participants: “*I almost wish that there were more opportunities to talk about burnout during the year. I would have really appreciated that.*” and “*Back in 1st and 2nd year the student affairs office had these wellness checks. I wish we had that in third year because we can all sign up for one but a lot of us are too busy to even think of it.”*

Participants appreciated protected time for resilience curricula and having a safe space to discuss resilience topics: “*[T] here was something special about having an opportunity to sort of vent about my experiences... I don’t feel like there’s anyone else in the program that I’d be willing to reveal that stuff to. And talking to each other is helpful, but it doesn’t have that degree of separation*” [105]. Similar trend of having a venue to openly discuss personal issues with peers was repeated in Pereira et al. (2015), Bird et al. (2017) and Tucker et al. (2017) as the participants felt the resilience curriculum provided a unique opportunity to build a community of shared experiences that allowed validation of participants’ worries and hardships. Understandably, 8% reported feeling vulnerable in sessions [98].

### Risk of bias assessment

To assess potential risk of bias, we used the ROBINS-I scoring system to evaluate bias across seven domains (Additional file 3): confounding bias, selection bias, classification bias, intervention bias, attrition bias, measurement bias, and reporting bias. Overall, the risk of bias was moderate for all studies, which, in light of the non-randomized nature of the studies and reliance on self-reporting measures, is understandable. While confounding bias and classification bias were deemed to be low in most of the included studies, selection bias was moderate in 9 (43%) and measurement bias was moderate in all (100%) studies. Due to the high attrition rate in most studies, bias due to missing data was low in 6 studies (29%), moderate in 12 studies (57%), and unknown in 3 studies (14%). To date, there have been only two randomized controlled trials that assessed the curricular efficacy of a resilience program, however these studies were assessed with the same bias scoring system to maintain coherence with other included studies.

## Discussion

In response to the growing awareness of the high risk of burnout and stress in health professionals, there has been growing interest in resilience in medical education over the past decade. While physicians as a group have significantly higher resilience than the general public [106], efforts to overcome systemic challenges in the clinical care environment are truly needed to curve physician burnout and foster well-being. To the best of our knowledge, this is the first systematic review to provide a comprehensive summary of published literature on the effect of resilience curricula in undergraduate and postgraduate medical education. Our results highlight the considerable heterogeneity in content, delivery, and outcomes of resilience curricula implemented to date in medical education. In particular, in spite of the modest improvement in resilience and well-being reported in many studies, a number of other studies [94, 95, 98] suggested that the implementation of the program resulted in detrimental effects to the participants’ overall resilience. Notably, all three studies delivered their curricula over 6 months to a year, and thus their negative results may have been confounded by an interaction between trainees’ resilience level and the progression or level of training. True to the significant heterogeneity seen in the resilience curricula in this review, currently no standardized, efficacy-proven resilience curriculum exists around the world that could be implemented by undergraduate and postgraduate medical programs. This present review clearly calls for the development of more systematic and evidence-based programs and resources which are desperately needed in the fight to reduce physician stress and burnout.

The strengths of this review include its systematic methodology; prospectively registered with a clearly defined objective, comprehensive and detailed search strategy, and the screening of each study’s risk of bias and methodological rigor. In spite of this, the findings of the present review should be interpreted in light of several limitations. Firstly, all included studies had moderate risk of bias according to the ROBINS-I tool for assessing the risk of bias in non-randomized studies. Therefore, the results presented in this review must be interpreted in light of these potential biases. Secondly, most of the included studies did not assess long-term resilience outcomes of resilience programs. As such, it remains unclear whether the resilience curricula successfully fostered lasting resilience in medical students and residents, or if the reported benefits were transient. As previous research has shown resilience is an acquired skill and thought pattern that requires continual practice to achieve mastery, an assessment methodology that incorporates long-term follow-up on curriculum participants should be considered in future studies. In parallel, resilience curricula may result in long-term resilience building by providing strategies that trainees can then choose to continuously implement throughout their training and future practice. By providing appropriate tools, long-term sustainable resilience may be achievable. Next, all studies relied on self-reporting psychometric instruments and, as such, it is unclear whether the reported changes in the indices of resilience curricula have meaningful objective implications for personal and professional development. Furthermore, although an increasing number of resilience curricula are being offered throughout UGME and PGME, the results of this review demonstrate vast heterogeneity of curricular methods, ranging from one-time didactic lectures to a mix of lecture-based and practical sessions over many months. Consequently, a standardized approach to resilience curricula development does not currently exist. However, the lack of a common foundation between resilience programs is not surprising given the early nature of resilience-centered initiatives. Additionally, the diversity of medical trainees and trainee stressors may necessitate a resilience curriculum tailored specifically to their unique beliefs, values, and stressors. The broad range of curricula makes inter-institutional comparison difficult. More research is necessary to determine whether a standardized approach to resilience is feasible and beneficial, or if medical programs should develop unique curricula adapted to their trainees.

Despite these limitations, this systematic review provides a guidance for future resilience curricula development and related studies. Future research on resilience in medical education should include clear operationalization of resilience and curricular components to enable reproducibility and accurate comparison of outcomes. In addition, it would be beneficial to compare conventional in-class delivery of resilience curricula, the most common mode of delivery among the included studies, with other educational methods such as online learning and electronic interventions (e.g., smartphone apps) to explore their efficacy in different populations and contexts. Future research must assess the effect of varying duration and frequency of resilience curricula on participants’ resilience and personal wellness; thereby elucidating the optimal curriculum delivery timeline. Lastly, future investigations must incorporate rigorous and robust research methodology to accurately determine the true effects of resilience training. A recent systematic review revealed that there is currently no gold standard outcome measure of resilience, thus rendering it difficult to assess criterion validity of various measures [107]. Further research should aim to develop a common instrument for objective resilience measurement.

The development and implementation of resilience education is a new phenomenon highlighted by an upstroke in publications during the past decade. Following recommendations by the ACGME in the United States and CanMEDS in Canada to improve trainee well-being, UGME and PGME programs in Canada and the US began to develop their own curricula to better support their trainees. Although medical trainee burnout has been extensively reported internationally, the implementation of resilience curricula has remained mostly limited to the US. Results of these preliminary studies can help to inform resilience education worldwide.

The benefits of resilience curricula are relevant to medical education, and at large, the medical workforce in today’s society. Longitudinal studies have responded to the growing concern of the highly prevalent burnout and stress in the medical field and have demonstrated the predictive value of self-report resilience scales on future mental health problems in their career [108]. Yet, there remains hesitancy and lack of enthusiasm for routine provision of resilience training in medicine, where the belief that “doctors are invincible” remains fixed [109]. It is time to address the stigma, recognize vulnerabilities and push for cultural change.

## Supplementary Information

**Additional file 1:** PRISMA checklist.

**Additional file 2:** Search strategy.

**Additional file 3:** ROBINS-I risk of bias assessment.

## Data Availability

All data generated or analyzed during this study are included in this published article and its supplementary information files.
