# Supplementary material for: Addressing the physician burnout epidemic with resilience curricula in medical education: a systematic review
Source: BMC Med Educ. 2021 Feb 1;21:80. doi: 10.1186/s12909-021-02495-0 (PMC7849619; doi:10.1186/s12909-021-02495-0)
Supplement: Supplementary file 2 — Additional file 2: Search strategy. [file 12909_2021_2495_MOESM2_ESM.docx]

**Additional file 2: Search strategy**

Searches performed June 16^h^, 2020

MEDLINE(R) ALL (Ovid, 1946 to June 15, 2020)

1. exp Education, Medical/

2. Students, Medical/

3. ((medical* or medicine?) adj2 (educat* or train* or student*)).ti,ab,kf.

4. (resident? or fellow* or intern?).ti,ab,kf.

5. (UGME* or PGME*).ti,ab,kf.

6. or/1-5

7. Resilience, Psychological/

8. Adaptation, Psychological/

9. resilien*.ti,ab,kf.

10. (cope or coping).ti,ab,kf.

11. ((positiv* or psychol*) adj2 (adapt* or adjust*)).ti,ab,kf.

12. ((withstand* or overcom* or resist* or recover* or thriv* or adapt* or adjust* or bounc* back) adj3 (stress* or trauma* or adversit*)).ti,ab,kf.

13. or/7-12

14. exp Curriculum/

15. (curricul* or course* or class or classes).ti,ab,kf.

16. Program Development/

17. Program Evaluation/

18. (program* or intervention* or workshop* or training).ti,ab,kf.

19. or/14-18

20. 6 and 13 and 19

21. (editorial or letter or comments).pt.

22. 20 not 21

Results: **2,230** citations retrieved

Embase (Ovid, 1947 to 2020 June 15)

1. exp medical education/

2. medical student/

3. ((medical* or medicine?) adj2 (educat* or train* or student*)).ti,ab,kw.

4. (resident? or fellow* or intern?).ti,ab,kw.

5. (UGME* or PGME*).ti,ab,kw.

6. or/1-5

7. psychological resilience/

8. exp psychological resilience scale/

9. coping behavior/

10. resilien*.ti,ab,kw.

11. (cope or coping).ti,ab,kw.

12. ((positiv* or psychol*) adj2 (adapt* or adjust*)).ti,ab,kw.

13. ((withstand* or overcom* or resist* or recover* or thriv* or adapt* or adjust* or bounc* back) adj3 (stress* or trauma* or adversit*)).ti,ab,kw.

14. or/7-13

15. education program/

16. curriculum/ or curriculum development/

17. workshop/

18. (curricul* or course* or class or classes).ti,ab,kw.

19. (program* or intervention* or workshop* or training).ti,ab,kw.

20. or/15-19

21. 6 and 14 and 20

22. abstract report/ or letter/

23. (Letter or Editorial or conference abstract).pt.

24. or/22-23

25. 21 not 24

Results: **2,472** citations retrieved

APA PsycInfo (Ovid, 1806 to June Week 2 2020)

1. exp medical education/

2. medical students/

3. ((medical* or medicine?) adj2 (educat* or train* or student*)).tw.

4. (resident? or fellow* or intern?).tw.

5. (UGME* or PGME*).tw.

6. or/1-5

7. "resilience (psychological)"/ or coping behavior/

8. resilien*.tw.

9. (cope or coping).tw.

10. ((positiv* or psychol*) adj2 (adapt* or adjust*)).tw.

11. ((withstand* or overcom* or resist* or recover* or thriv* or adapt* or adjust* or bounc* back) adj3 (stress* or trauma* or adversit*)).tw.

12. or/7-11

13. curriculum/ or curriculum based assessment/ or curriculum development/

14. (curricul* or course* or class or classes).tw.

15. educational programs/

16. exp program development/ or exp program evaluation/

17. (program* or intervention* or workshop* or training).tw.

18. or/13-17

19. 6 and 12 and 18

20. (Book or Authored Book or Edited Book or Dissertation Abstract or Encyclopedia).pt.

21. 19 not 20

Results: **938** citations retrieved

ERIC (Ovid, 1965 to March 2020)

1. medical education/ or graduate medical education/

2. medical students/

3. ((medical* or medicine?) adj2 (educat* or train* or student*)).tw.

4. (resident? or fellow* or intern?).tw and (medicine* or medical* or clinical*).tw.

5. (UGME* or PGME*).tw.

6. or/1-5

7. "resilience (psychology)"/ or coping/

8. resilien*.tw.

9. (cope or coping).tw.

10. ((positiv* or psychol*) adj2 (adapt* or adjust*)).tw.

11. ((withstand* or overcom* or resist* or recover* or thriv* or adapt* or adjust* or bounc* back) adj3 (stress* or trauma* or adversit*)).tw.

12. or/7-11

13. curriculum/ or core curriculum/ or courses/ or integrated curriculum/ or course content/ or curriculum design/ or curriculum development/ or curriculum enrichment/ or curriculum evaluation/ or curriculum research/

14. curriculum implementation/

15. program development/ or program administration/ or program design/ or program effectiveness/ or program evaluation/ or program implementation/ or program improvement/ or programs/

16. (curricul* or course* or class or classes).tw.

17. workshops/

18. (program* or intervention* or workshop* or training).tw.

19. or/13-18

20. 6 and 12 and 19

Results: **146** citations retrieved

Education Source (EBSCOHost)

| \| **#** \| **Query** \| **Results** \| \| --- \| --- \| --- \| \| S1 \| DE "Medical education" OR DE "Clinical clerkship" OR DE "Clinical medical education" OR DE "Family medicine education" OR DE "Graduate medical education" OR DE "Medical students" \| 17,910 \| \| S2 \| TI ( ((medical* or medicine?) N2 (educat* or train* or student*)) ) OR AB ( ((medical* or medicine?) N2 (educat* or train* or student*)) ) \| 24,225 \| \| S3 \| TI ( (resident? or fellow* or intern?) and (medicine* or medical* or clinical*) ) OR AB ( (resident? or fellow* or intern?) and (medicine* or medical* or clinical*) ) \| 2,916 \| \| S4 \| TI ( (UGME* or PGME*) ) OR AB ( (UGME* or PGME*) ) \| 24 \| \| S5 \| S1 OR S2 OR S3 OR S4 \| 32,910 \| \| S6 \| (DE "Resilience (Personality trait)") OR (DE "Psychological adaptation") \| 13,080 \| \| S7 \| TI ( resilien* or cope or coping ) OR AB ( resilien* or cope or coping ) \| 36,084 \| \| S8 \| TI ( ((positiv* or psychol*) N2 (adapt* or adjust*)) ) OR AB ( ((positiv* or psychol*) N2 (adapt* or adjust*)) ) \| 2,671 \| \| S9 \| TI ( ((withstand* or overcom* or resist* or recover* or thriv* or adapt* or adjust* or bounc* back) N3 (stress* or trauma* or adversit*)) ) OR AB ( ((withstand* or overcom* or resist* or recover* or thriv* or adapt* or adjust* or bounc* back) N3 (stress* or trauma* or adversit*)) ) \| 2,211 \| \| S10 \| S6 OR S7 OR S8 OR S9 \| 45,173 \| \| S11 \| DE "Curriculum planning" OR DE "Curricula (Courses of study)" OR DE "Instructional systems design" OR DE "Curriculum alignment" OR DE "Curriculum change" OR DE "Understanding by design (Curriculum planning)" \| 111,096 \| \| S12 \| (((DE "Course content (Education)" OR DE "Course evaluation (Education)" OR DE "Course outlines (Education)") OR (DE "Educational programs" OR DE "Program design (Education)")) OR (DE "Educational programs -- Evaluation")) OR (DE "Program development (Education)" OR DE "Program effectiveness (Education)" OR DE "Program implementation (Education)" OR DE "Program improvement (Education)" OR DE "Program validation (Education)") \| 28,584 \| \| S13 \| TI ( curricul* or course* or class or classes or program* or intervention* or workshop* or training ) OR AB ( curricul* or course* or class or classes or program* or intervention* or workshop* or training ) \| 1,064,166 \| \| S14 \| S11 OR S12 OR S13 \| 1,093,949 \| \| S15 \| S5 AND S10 AND S14 \| 321 \| |
| --- | --- | --- | --- | --- | --- | --- | --- | --- | --- | --- | --- | --- | --- | --- | --- | --- | --- | --- | --- | --- | --- | --- | --- | --- | --- | --- | --- | --- | --- | --- | --- | --- | --- | --- | --- | --- | --- | --- | --- | --- | --- | --- | --- | --- | --- | --- | --- | --- |

Results: **315** citations retrieved

The total number of references retrieved before duplicate records removed is **6,101**. Using Covidence’s automatic duplicate removal feature, 2,101 duplicate records were removed. Which leaves **4,000** **references** for the screening phase.
