# Supplementary material for: Addressing the physician burnout epidemic with resilience curricula in medical education: a systematic review
Source: BMC Med Educ. 2021 Feb 1;21:80. doi: 10.1186/s12909-021-02495-0 (PMC7849619; doi:10.1186/s12909-021-02495-0)
Supplement: Supplementary file 3 — Additional file 3: ROBINS-I risk of bias assessment. [file 12909_2021_2495_MOESM3_ESM.docx]

**Additional file 3: ROBINS-I risk of bias assessment**

| **Reference** | **Bias due to confounding** | **Bias in selection of participants into the study** | **Bias in classification of interventions** | **Bias due to deviations from intended interventions** | **Bias due to missing data** | **Bias in measurement of outcomes** | **Bias in selection of the reported result** | **Overall risk of bias (low / moderate / serious / critical)** |
| --- | --- | --- | --- | --- | --- | --- | --- | --- |
| Kelly et al., 1982 | low | moderate | low | ? | ? | moderate | ? | moderate |
| Holtzworth-Munroe et al., 1985 | low | moderate | low | ? | moderate | moderate | low | moderate |
| McCue et al., 1991 | low | moderate | low | ? | low | moderate | low | moderate |
| Saadat et al., 2012 | low | low | low | low | low | moderate | low | moderate |
| Peng et al., 2014 | low | moderate | low | low | ? | moderate | ? | moderate |
| Pereira et al., 2015 | low | moderate | low | ? | low | moderate | moderate | moderate |
| Brennan et al., 2016 | moderate | moderate | low | ? | moderate | moderate | low | moderate |
| Rogers, 2016 | low | moderate | low | ? | moderate | moderate | low | moderate |
| Runyan et al., 2016 | low | low | low | ? | low | moderate | low | moderate |
| Bird et al., 2017 | low | low | low | ? | moderate | moderate | low | moderate |
| Dyrbye et al., 2017 | low | low | low | low | moderate | moderate | low | moderate |
| Slavin et al., 2017 | low | low | low | ? | low | moderate | low | moderate |
| Tucker et al., 2017 | low | moderate | low | ? | moderate | moderate | ? | moderate |
| Chaukos et al., 2018 | low | low | low | low | moderate | moderate | low | moderate |
| Riall et al., 2018 | low | low | low | low | moderate | moderate | low | moderate |
| Brennan et al., 2019 | low | low | low | ? | moderate | moderate | low | moderate |
| Orr et al., 2019 | low | low | low | low | moderate | moderate | moderate | moderate |
| Saint Martin et al., 2019 | low | low | low | ? | moderate | moderate | low | moderate |
| Shapiro et al., 2019 | moderate | moderate | low | ? | low | moderate | low | moderate |
| Forbes et al., 2020 | low | low | low | ? | moderate | moderate | low | moderate |
| Song et al., 2020 | low | low | low | ? | moderate | moderate | moderate | moderate |
